# MiRNA-195-5p Functions as a Tumor Suppressor and a Predictive of Poor Prognosis in Non-small Cell Lung Cancer by Directly Targeting CIAPIN1

**DOI:** 10.1007/s12253-018-0552-z

**Published:** 2019-01-12

**Authors:** Jing Zheng, Tingting Xu, Feng Chen, Ying Zhang

**Affiliations:** Department of Respiratory Medicine, Taizhou Hospital, 381 East Zhongshan Road, Jiaojiang District, Taizhou, Zhejiang, 318000 NO China

**Keywords:** Non-small cell lung cancer, miR-195-5p, Overall survival, Prognosis, CIAPIN1

## Abstract

Accumulating evidence suggests that microRNAs (miRNAs) has been proven to be a critical regulator in the tumor progression, of which miR-195-5p was reported to function as tumor suppressor in prostate cancer and oral squamous cell carcinoma. However, studies on the clinical significance and biological function of miR-195-5p in non-small cell lung cancer (NSCLC) were still unavailable. Here, we reported that the expression of miR-195-5p was decreased in NSCLC tissues and cell lines. Downregulation of miR-195-5p was significantly associated with TNM stage, tumor size and lymph node metastasis. The Kaplan-Meier survival analysis demonstrated that the survival time of NSCLC patients with high expression of miR-195-5p was longer than those with low expression during the 5-year follow up period (*p* = 0.0410). COX regression analysis indicated that miR-195-5p expression was an independent prognostic indicator for the survival of NSCLC patients (HR = 2.45, 95% CI: 1.53–4.63; *p* = 0.007). Results of functional analyses revealed that overexpression of miR-195-5p in A549 cells inhibited cell proliferation, induced cell cycle G0/G1 phase arrest and apoptosis using MTT and flow cytometry analysis. Furthermore, bioinformatics and luciferase reporter assays demonstrated that cytokine-induced apoptosis inhibitor 1 (CIAPIN1), an anti-apoptotic molecule was a direct target of miR-195-5p in NSCLC cells. Meta-analysis based on Oncomine database showed CIAPIN1 was significantly up-regulated in human lung cancer tissues. Consistently, knockdown of CIAPIN1 phenocopied the inhibitory effects of miR-195-5p overexpression in NSCLC cell function. These findings suggest that miR-195-5p could be used as a potential prognostic predictor and tumor suppressor in NSCLC.

## Introduction

Non-small-cell lung cancer (NSCLC) is considered as the major subtype of lung cancer with accounting for 85%–90% of all cases [1]. Persistent cough, pain, and weight loss are common symptoms in patients with NSCLC [2]. Despite great advances in surgery, adjuvant therapy, stereotactic radiotherapy, the 5-year overall survival (OS) rate of NSCLC patients remains very poor [3]. Therefore, optimizing current treatment methods requires a deep understanding of the pathogenesis of NSCLC.

MicroRNAs are short (~20–23 nucleotide) non-coding RNAs that usually expressed in a variety of tissues and cell types, and mediates post-transcriptional gene silencing in mammals through interaction with their target mRNAs [4]. Accumulating reports of the biological behaviors of miRNAs in development, proliferation, apoptosis, and differentiation have increased the academia’s awareness of the important element of miRNAs in the pathophysiology of human disease, including cancer [5]. Recent key miRNAs including miR-21 [6], miR-451 [7], miR-126 [8], and miR-30a [9] have been shown to be deregulated in NSCLC and play a key role in cancer progression and metastasis. Down-regulation of miR-375 was found to be associated with advanced NSCLC stage and lymphatic metastasis [10]. It has been shown that miR-195-5p is increased in gemicitabine-resistant NSCLC cells provides the first suggestion of miR-195-5p may be associated with cellular response to drug treatment [11]. Besides, novel diagnostic value of serum miR-195-5p and its role as a prognostic factor was revealed in NSCLC cancer [12]. Moreover, miR-195 has been reported to suppress tumorigenesis of NSCLC though modulating cyclin D3 and surviving [13]. However, the measurement and clinical significance of miR-195-5p in NSCLC remains undefined.

The cytokine-induced apoptosis inhibitor-1 (CIAPIN1), is known as an anti-apoptotic molecule which shows no sequence homology to a series of apoptosis molecules including Bcl-2 and caspase family members [14]. It is a key mediator of RAS signaling pathways, mediates maintenance of hematopoiesis in the fetal liver [15]. Interestingly, CIAPIN1 has emerged as a candidate indicator for diagnosis, prognosis and therapeutic target in multiple human cancers [16]. Based on the analysis of Hao et al. [17], CIAPIN1 was found to confer multidrug resistance in gastric cancer cells though elevating MDR-1 and MRP-1 profiles. Enhanced expression of CIAPIN1 resulted in suppression of clear renal cell carcinoma-derived cells G1-phase arrest as revealed by decreased levels of cyclin (D1, E), CDK (2,4), p-Rb, and VEGF, as well as increased levels of p27^Kip1^ [18]. Moreover, cancerous lung carcinoma tissues showed lower positive rate of CIAPIN1 as compared to that in the than that in the noncancerous tissues [19].

Considering the above observations, we hypothesized that miR-195-5p might play an important role in NSCLC by targeting CIAPIN1. Thus, in the present study, we aimed to explore the molecular mechanism by which miR-195-5p mediates the growth and proliferation of NSCLC cells through its targeting of CIAPIN1.

## Materials and Methods

### Patients and Tissue Specimens

A total of 60 pairs of tumor samples and adjacent lung tissue samples were collected from NSCLC patients who underwent surgical resection at the Department of Respiratory Medicine, Taizhou Hospital (Zhejiang, China) in the study. No patients had undergone preoperative chemotherapy or radiotherapy. The patients’ medical records, follow-up data and histopathological diagnoses were fully documented. The medical records included age, gender, clinical stage, differentiation, lymph node metastasis, smoking status, and overall survival (OS) time. Survival time was calculated in months from the day of resection until death, or censored if no death was noted at follow-up date. All patients gave informed consent before tissue collection and all resected lung tissues were immediately stored in liquid nitrogen at −80 °C before use. This study was approved by the Ethical Review Committee of Taizhou Hospital (Zhejiang, China).

### Cell Culture and Transfection

Human NSCLC cell lines, H1299, 95D, H1650, and A549, as well as immortalized human bronchial epithelial cell BEAS-2B were purchased from the American Type Culture Collection (ATCC, Manassas, VA, USA). H1299, 95D and H1650 cells were cultured in RPMI-1640 medium (Gibco, NY, USA). A549 and BEAS-2B cell lines were cultivated in Dulbecco’s Modified Eagle’s Medium (DMEM, Gibco, NY, USA). All media were supplemented with 10% fetal bovine serum (FBS, Gibco, Grand Island, NY, USA), 100 U/mL penicillin, and 100 μg/mL streptomycin (Gibco). All cell lines were maintained in a humidified incubator containing 5% CO_2_ at 37 °C.

Before transfection, A549 cells were seeded into six-well plates at a density of 1 × 10^5^ cells per well and incubated overnight. The miR-195-5p mimics, small interfering RNA for CIAPIN1 (siCIAPIN1) and their corresponding negative control (miR-NC and siNC, respectively) were chemically synthesized by Shanghai GenePharma Co., Ltd. (Shanghai, China). When the cells reached 70–80% confluence, they were transfected with 100 nmol of above oligonucleotides using Lipofectamine 2000 (Invitrogen, Carlsbad, CA, USA) according to the manufacturer’s protocol. Then transfected cells were further analysis after incubated for 48 h at 37 °C.

### Quantitative Real-Time (qRT-PCR)

For miR-195-5p expression detection, all miRNAs were extracted from tissues and cell lines using the miRNeasy mini kit (Qiagen, Cat. 217,004, Hilden, Germany) and Reverse transcription was performed using the TaqMan MiRNA reverse transcription kit (Applied Biosystems) according the manufacturer’s instructions. The expression levels of miR-195-5p were quantified using TaqMan miRNA assays (Applied Biosystems) with U6 as internal control. For quantitative detection of CIAPIN1, complementary DNA was synthesized using PrimeScript™ RT reagent kit (TaKaRa, Dalian, China). Then CIAPIN1 mRNA was detected by SYBR Green Kit (TaKaRa, Dalian, China) with GAPDH as internal control. All qRT-PCR analysis was performed using an Applied Biosystems 7900HT Fast Real-Time PCR System instrument (Applied Biosystems). The primer sequences were listed as followed: 5′-GGGGTA GCAGCACAGAAAT-3′ (forward) and 5′-TCCAGTGC GTGTCGTGGA-3′ (reverse) for miR-195-5p; 5′-TG CGGGTGCTCGCTTCGCAGC-3′ (forward) and 5′-CCA GTGCAGGGTCCGAGGT-3′ (reverse) for U6; 5′-CACCAAGAAGTCTTCTCCTTCAGTG-3′ (forward) and 5′-GCTGAGAGGGTCCACAGCT-3′ (reverse) for CIAPIN1; 5’-GGAGCGAGATCCCTCCAAAAT-3′ (forward) and 5’-GGCTGTTGTCATACTTCTCATGG-3′ (reverse) for GAPDH. Each measurement was performed in triplicate. The 2^−ΔΔCt^ method was used to determine the relative quantitation of miR-195-5p and CIAPIN1 mRNA expression.

### OS Analysis Using Kaplan–Meier Method

Total 60 cases of NSCLC cases were divided into higher-than-median group (*n* = 22) and lower-than-median group (*n* = 38) according to the median miR-195-5p expression value used as a cutoff. The value of ‘0’ was entered for a patient alive event (live) and ‘1’ was entered in the event of death. The survival curve was drawn using the Graphpad Prism 6 Project, and the differences between the two groups were evaluated using the Kaplan-Meier method with log-rank test.

### Cell Proliferation Assays

Cell proliferation was determined using the MTT assay according to manufacturer’s instructions. In brief, transfected A549 cells were seeded into a 96-well plate at a density of 3 × 10^3^ cells per well and incubated at 37 °C for different time periods (1, 2, 3, 4, and 5 day, respectively). Then 10 μL MTT reagent (0.5 mg/mL, (Sigma-Aldrich, St. Louis, Mo, USA) were added to the culture medium. After incubation for 2 h, the supernatant was removed and the crystals were resolved by adding 150 μL DMSO (Sigma-Aldrich). The optical density (OD) of each well was measured at 595 nm using a microplate reader (SpectraMax M5, Molecular Devices, CA, USA).

### Cell Cycle and Apoptosis Assays

For cell cycle analysis, transfected A549 cells were harvested, washed three times with cold PBS and fixed with cool 70% ethanol at room temperature. Then the cells were stained with 50 μg/ml propidium iodide (PI, BD Biosciences, CA, USA) following the manufacturer’s protocol for 30 min, followed by cell cycle analysis using a FACS Calibur Flow Cytometer (Beckman Coulter, Atlanta, GA, USA). To measure cell apoptosis, Annexin V-FITC apoptosis detection kit (BD Biosciences, CA, USA) was applied. Briefly, cells were collected, washed twice with cold PBS, and re-suspended in Annexin V-binding buffer. Following incubation with Annexin V-FITC and PI for 15 min in the dark, the early apoptosis rate (Annexin V+/PI-) and late apoptosis rate (Annexin V+/PI+) were analyzed using a FACS Calibur Flow Cytometer (Beckman Coulter, Atlanta, GA, USA).

### Bioinformatics Predication and Dual Luciferase Reporter Assay

Publicly available algorithms (TargetScan, miRanda, miRwalk) were used to predict the potential targets of miR-195-5p in humans. CIAPIN1 was selected as a potential target of miR-195-5p by the three algorithms. The 3’UTR of the CIAPIN1-containing miR-195-5p binding sites and the mutated sequences that interacted with the seed sequence of miR-195-5p were synthesized by GenePharma Co., Ltd. (Shanghai, China), which were sub-cloned into the dual-luciferase reporter gene vector psicheck-2 (Promega, Madison, WI, USA) to construct the recombinant wild type reporter gene vector WT-CIAPIN1–3′ UTR and MUT-CIAPIN1–3′ UTR, respectively. For the dual luciferase assay, A549 cells (5000 cells per well) were seeded in 96-well plates and transfected with 25 ng luciferase reporter gene vector containing WT-CIAPIN1–3′ UTR or MUT-CIAPIN1–3′ UTR together with 50 ng miR-195-5p mimics or miR-NC using Lipofectamine 2000 (Invitrogen, USA) according to the manufacturer’s instructions. Forty-eight hours after transfection, the cells were harvested and analyzed for luciferase activity using the Dual-Luciferase Reporter Assay System (Promega, USA).

### Oncomine Database Analysis

A meta-analysis on online Oncomine Expression Array database (www.oncomine.org) was performed to evaluate the different expression of CIAPIN1 between lung cancer and normal tissues. Through searching the following terms: “CIAPIN1”, “Cancer vs. Normal Analysis”, “lung cancer vs. normal tissues” and “mRNA”, total seven datasets were screened including Selamat Lung [20], Garber Lung [21], Hou Lung [22], Landi Lung [23], Okayama Lung [24], Su Lung [25] and Wachi Lung [26] datasets. In addition, three of these seven datasets, including Selamat Lung [20], Landi Lung [23] and Okayama Lung [24] were extracted and analyzed for the different expression of CIAPIN1 between NSCLC and normal lung tissues by GraphPad Prism 6.0.

### Protein Extraction and Western Blotting

The proteins were extracted from the transfected cells using RIPA lysis buffer containing 1% protease inhibitor (Sigma, St Louis, MO). Supernatant protein concentration was determined using a BCA Quantification Kit (Beyotime, Beijing, China). Equal amounts of protein samples were separated by 12% SDS-PAGE and transferred to a PVDF membrane (Millipore, Hercules, CA, USA). The membrane was then blocked with 5% skimmed milk in Tris-buffered saline containing 0.1% Tween-20 (TBST) for 2 h at room temperature, followed by incubation with primary antibodies against CIAPIN1 (cat. no. ab154904, Abcam) and GAPDH (cat. no. sc-47,724; Santa Cruz Biotechnology, Inc.) overnight. After washing with TBST three times, the membrane was incubated with horseradish peroxidase-conjugated secondary antibody (cat. no. sc-2005; Santa Cruz Biotechnology, Inc.) for 2 h. Finally, the immunoreactive bands were visualized using the ECL Western Blotting kit (Pierce; Thermo Fisher Scientific, Inc.), using GAPDH as a loading control.

### Statistical Analysis

Data are expressed as the mean ± standard deviation (SD) of triplicate for each experiment. Statistical analysis was performed using SPSS 17.0 (SPSS, Inc., Chicago, IL, USA). The Chi-square test was performed to determine the relationship between miR-195-5p expression and clinicopathological parameters. Survival was estimated using the Kaplan-Meier method, and the differences in survival according to miR-195-5p expression were compared using the log-rank test. Differences were evaluated using Student’s t test or one-way analysis of variance. The statistical significance level was accepted when a *p* value of less than 0.05.

## Results

### MiR-195-5p Was Significantly Down-Regulated and Correlated with Poor Prognosis in NSCLC Patients

**Fig. 1:**
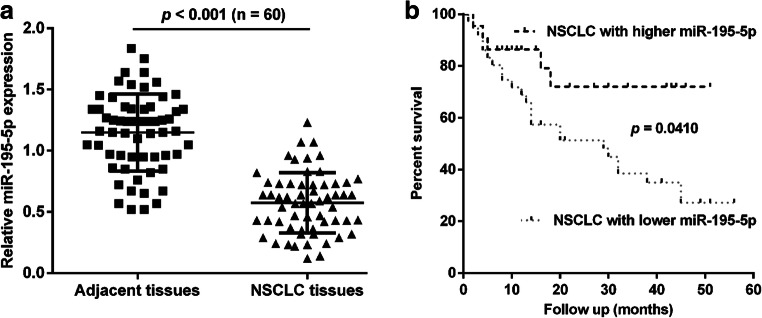
Downregulation of miR-195-5p correlated with poor prognosis of NSCLC. (**a**) Relative expression of miR-195-5p in NSCLC samples (*n* = 60) and adjacent non-cancerous tissues (n = 60) was measured by quantitative real-time PCR. (**b**) Kaplan-Meier method was evaluate the five-year survival rate of the patients with high (*n* = 22) and low (*n* = 38) expression of miR-195-5p

At first, the expression of miR-195-5p was determined in NSCLC tissues using qRT-PCR. Results revealed that miR-195-5p expression was significantly decreased in 60 pairs of human NSCLC tissues, in comparison with the corresponding adjacent tissues (Fig. 1a *p* < 0.001). In addition, the relationship between miR-195-5p and several clinicopathological features were investigated in NSCLC patients. These 60 NSCLC patients were divided into lower expressing miR-195-5p group (*n* = 38) and higher expressing miR-195-5p group (*n* = 22) according to the median miR-195-5p expression value used as a cutoff. Using the Chi-square analysis, we found decreased miR-195-5p expression was significantly correlated with tumor size (*p* = 0.031), TNM stage (*p* = 0.012) and lymph node metastasis (*p* = 0.006) (Table 1). Results of Kaplan-Meier analysis showed that the 5-year OS rates for NSCLC patients with low expression of miR-195-5p significantly reduced, when compared with the patients with high level of miR-195-5p (Fig. 1b, *p* = 0.0410). We next performed Cox’s univariate and multivariate hazard regression model to identify the independent factors that were significantly associated with OS and found tumor size (HR = 1.87, 95% CI: 1.25–2.79; *p* = 0.021), TNM stage (HR = 2.04, 95% CI: 1.74–2.86; *p* = 0.008) and miR-195-5p expression level (HR = 2.45, 95% CI: 1.53–4.63; *p* = 0.007) were recognized as significant prognostic factor for overall survival of NSCLC patients (Table 2). These findings suggest that decreased miR-195-5p expression might play a potential role in promoting malignant progression of NSCLC.

**Table 1:** Correlations of the miR-195-5p expression with clinicopathological factors in NSCLC (n = 60)

Patient characteristics	Cases (n = 60)	Expression of miR-195-5p		*P* value
		Low (n = 38)	High (n e= 22)	(chi-square test)
Gender				0.213
Male	48	29	19	
Female	12	9	3	
Age				0.115
< 60	29	17	12	
≥ 60	31	21	10	
Smoking history				0.204
Yes	43	23	20	
No	17	15	2	
Tumor size(cm)				0.031^*^
< 4	27	11	16	
≥ 4	33	27	6	
Tumor differentiation				0.701
Well/moderate	40	21	19	
Poor	20	17	3	
Histological type				0.058
ADC	27	15	12	
SCC	18	10	8	
Others	15	13	2	
TNM stage				0.012^*^
I + II	46	27	19	
III + IV	14	11	3	
Lymph node metastasis				0.006^*^
Negative	31	16	15	
Positive	29	22	7	

*P < 0.05; *NSCLC* non-small cell lung cancer; *miR* microRNA; *ADC* adenocarcinoma; *SCC* squamous cell carcinoma; *TNM* tumor-node-metastasis classification system

**Table 2:** Prognostic value of miR-195-5p expression for factors influencing overall survival of NSCLC patients in univariate and multivariate analyses by Cox regression

	Univariate analysis		Multivariate analysis	
Clinical pathologic parameters	HR (95%CI)	P value	HR (95%CI)	P value
Gender	1.08 (0.97–1.19)	0.237	1.05 (0.96–1.09)	0.534
Age	0.87 (0.56–1.21)	0.345	1.13 (0.81–1.82)	0.265
Smoking history	1.24 (0.78–1.52)	0.125	1.13 (0.79–1.76)	0.265
Tumor size (cm)	1.72 (1.17–2.34)	**0.004**	1.87 (1.25–2.79)	**0.021**
TNM stage	1.87 (1.48–2.63)	**0.011**	2.04 (1.74–2.86)	**0.008**
Tumor differentiation	0.84 (0.54–1.13)	0.134	1.21 (1.04–2.04)	0.189
Histological type	0.98 (0.59–1.34)	0.245	1.13 (0.83–1.84)	0.165
Lymph node metastasis	1.16 (0.85–1.354)	**0.031**	1.41 (1.02–1.96)	0.352
miR-195-5p expression	2.25 (1.58–2.47)	**0.013**	2.45 (1.53–4.63)	**0.007**

*HR* hazard ratio; *CI* confidence interval; *TNM* tumor-node-metastasis classification system

### Overexpression of miR-195-5p Significantly Suppressed Cell Proliferation, Promoted Cell Cycle Arrest and Apoptosis in NSCLC

**Fig. 2:**
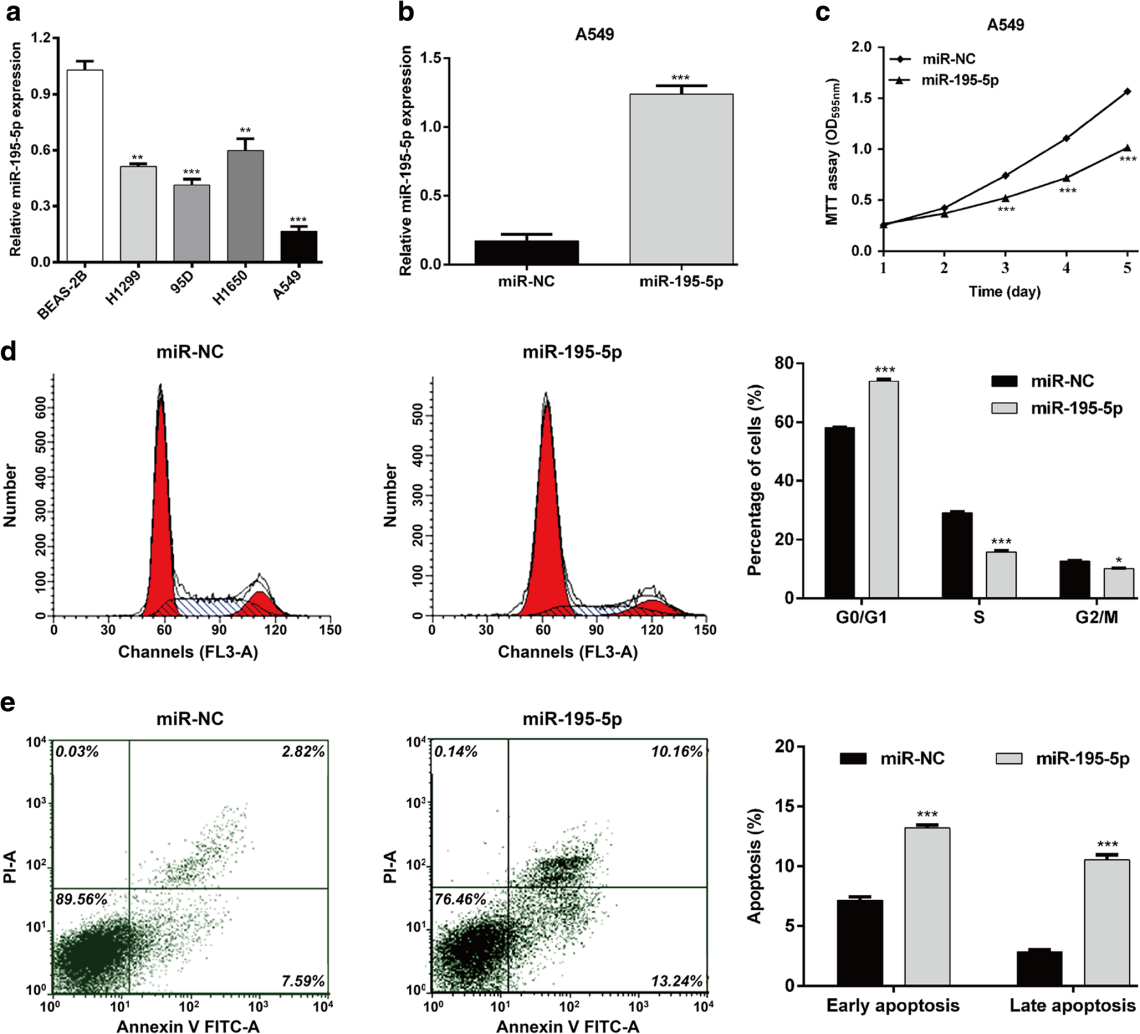
Effects of miR-195-5p overexpression on cell proliferation, cell cycle progression and apoptosis in NSCLC cells. **a** Expression of miR-195-5p in four NSCLC cell lines and normal bronchial epithelial cell BEAS-2B was determined by qRT-PCR. A549 cells were transfected with miR-195-5p or miR-NC for 48 h, respectively. **b** The expression of miR-195-5p was measured by quantitative real-time PCR. **c** Cell proliferation, (**d**) cell cycle distribution and (**e**) apoptosis were determined using MTT assay, flow cytometry with PI staining and flow cytometry with Annexin V/PI double staining assay, respectively in A549 cells. All values are represented as mean ± SD of three replicates. *p < 0.05, **p < 0.01, ***p < 0.001 compared with BEAS-2B cells, or miR-NC group

Considering the correlation between miR-195-5p expression and NSCLC progression, we further investigated the biological function of miR-195-5p in NSCLC in vitro. Using qRT-PCR assay, we first compared the expression of miR-195-5p in several NSCLC cell lines and normal bronchial epithelial cell BEAS-2B. As shown in Fig. 2a, the expression level of miR-195-5p was remarkably decreased in all the analyzed NSCLC cell lines (H1299, 95D, H1650, and A549), in comparison with BEAS-2B, among which, A549 presented the lowest miR-195-5p expression, thus used for the gain-of-function analysis. A549 cells were firstly transfected with miR-195-5p or miR-NC, and then confirmed the miR-195-5p expression. As expected, the expression of miR-195-5p was significantly overexpressed after miR-195-5p transfection in A549 cells (Fig. 2b, *p* < 0.001). Subsequently, MTT assay indicated that upregulation of miR-195-5p in A549 cells inhibited cell proliferation (Fig. 2c, *p* < 0.001). Moreover, results of flow cytometry analysis revealed that overexpression of miR-195-5p significantly induced cell cycle G0/G1 phase arrest, as confirmed elevated G0/G1 phase population (*p* < 0.001) and accordingly decreased S (*p* < 0.001) and G2/M phase (*p* < 0.05) population in A549 cells (Fig. 2d). Furthermore, flow cytometry analysis also demonstrated that overexpression of miR-195-5p promoted cell early and late apoptosis in A549 cells (Fig. 2e, *p* < 0.001). Therefore, these results demonstrated that miR-195-5p played a suppressive role in regulation of cell proliferation in NSCLC cells.

### CIAPIN1 Was a Direct Target of miR-195-5p in NSCLC

**Fig. 3:**
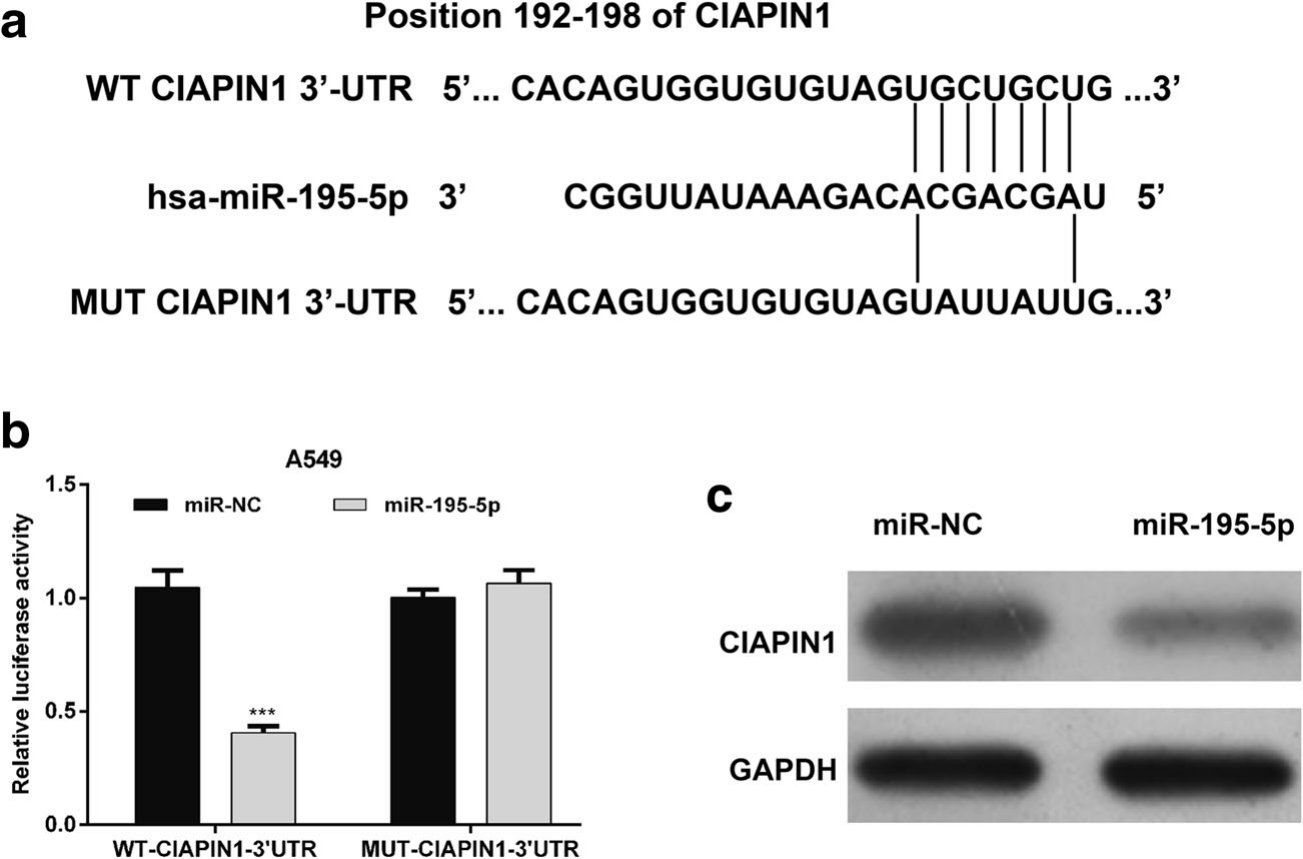
CIAPIN1 was a direct target of miR-195-5p in NSCLC. **a** Diagrams show the miR-195-5p putative binding sites and corresponding mutant sites of CIAPIN1. **b** miR-195-5p overexpression significantly suppressed the luciferase activity of CIAPIN1 containing a wild-type (WT) of 3′-UTR but not a mutant (MUT) 3′-UTR in A549 cells. All values are represented as mean ± SD of three replicates. ***p < 0.001 compared with miR-NC group; (**c**) miR-195-5p overexpression reduced the expression of CIAPIN1 protein in A549 cells

To further investigate the underlying mechanisms for how miR-195-5p exerts its functional effects on NSCLC cells, publicly available algorithms (TargetScan, miRanda, miRwalk) were used to predict the target genes according to consensus miR-195-5p binding sites. As shown in Fig. 3a, a sequence located at bases 192–198 of the CIAPIN1 3’-UTR was highly complementary with the seed sequence of miR-195-5p. Then a luciferase assay was performed to further confirm CIAPIN1 as a direct target of miR-195-5p. The results demonstrated that overexpression of miR-195-5p significantly decreased luciferase activity of WT construct of CIAPIN1 3’UTR, while no significant change of the luciferase activity was found with MUT construct of CIAPIN1 3’UTR (Fig. 3b, *p* < 0.001). Moreover, the relationship between miR-195-5p and CIAPIN1 was analyzed using western blot assay. As illustrated in Fig. 3c, overexpression of miR-195-5p significantly decreased protein levels of CIAPIN1 in A549 cells. Taken together, our data suggested that CIAPIN1 could be a direct downstream target of miR-195-5p in NSCLC cells.

### CIAPIN1 Was Significantly Upregulated in Lung Cancer Tissues by Meta-Analysis Based on Oncomine Database

**Fig. 4:**
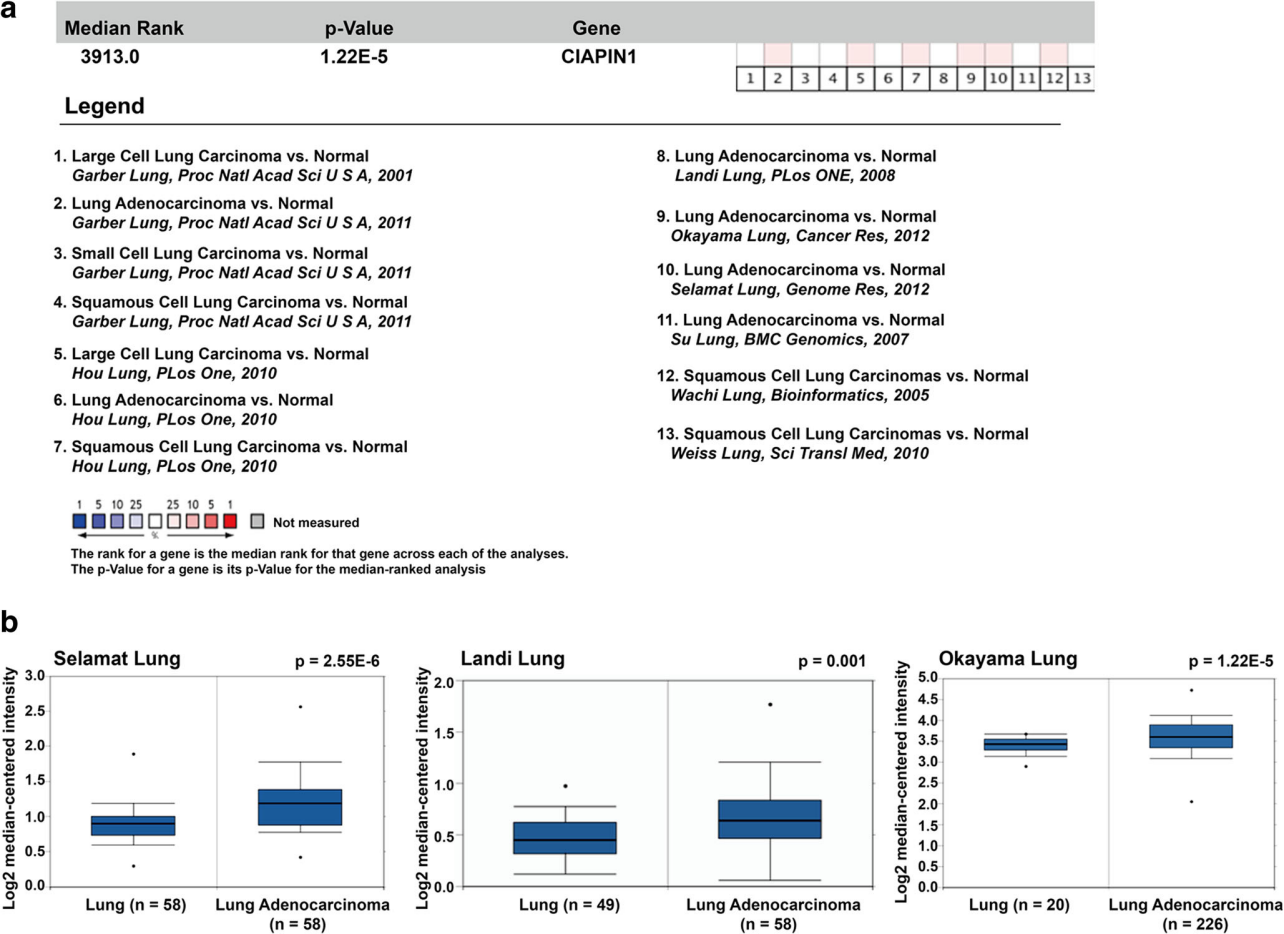
CIAPIN1 mRNA expression in lung cancer tissues vs. normal lung tissues was analyzed by using Oncomine microarray database. **a** Total seven microarray datasets regarding CIAPIN1 mRNA expression in lung cancer tissues vs. normal were included in our meta-analysis. Data are shown as the median rank of CIAPIN1 through each dataset analysis. *P* value for CIAPIN1 was presented using the median ranked analysis. **b** Three datasets, including Selamat Lung, Landi Lung and Okayama Lung were extracted for analyzing CIAPIN1 mRNA expression in NSCLC tissues vs. normal and data were presented as Log2 median-centered intensity

As CIAPIN1 was a potential target gene of miR-195-5p, we further investigated the expression of CIAPIN1 in lung cancer tissues by performing meta-analysis using public microarray datasets from Oncomine database. As shown in Fig. 4a, a total of seven online microarray datasets were included in our study. Meta-analysis of these datasets collectively revealed that increased CIAPIN1 mRNA expression was associated with lung cancer, including small cell lung carcinoma, large cell lung carcinoma, squamous cell lung carcinoma and lung adenocarcinoma as compared with normal lung tissues (gene median rank: 3913.0, *p* = 1.22E-5). We also showed increased CIAPIN1 mRNA expression in lung adenocarcinoma tissues compared with normal tissues by selecting three datasets (Fig. 4b). These analyses suggested that CIAPIN1 might play a potential carcinogenesis in lung cancer.

### Knockdown of CIAPIN1 Phenocopied the Inhibitory Effects of miR-195-5p Overexpression in NSCLC

**Fig. 5:**
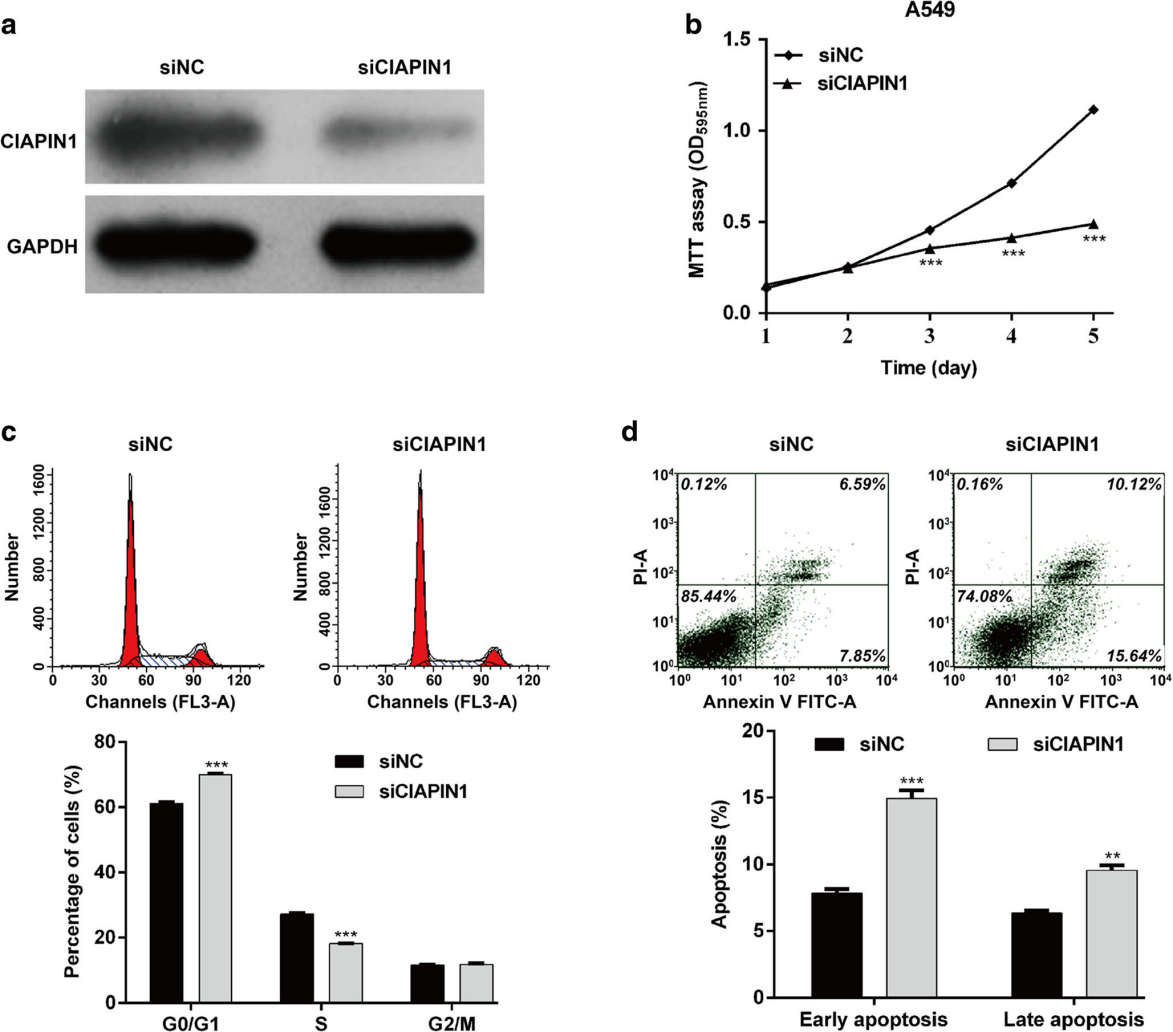
Knockdown of CIAPIN1 phenocopied the inhibitory effects of miR-195-5p overexpression in NSCLC. A549 cells were transfected with siCIAPIN1 or siNC for 48 h. **a** The expression of CIAPIN1 protein was determined by western blot analysis. **b** Cell proliferation was determined using MTT assay. **c** Flow cytomery was performed to detect cell cycle distribution and the percentage of cells at G0/G1, S and G2/M phase was calculated. **d** Flow cytometry was used to detect cell apoptosis. All values are represented as mean ± SD of three replicates. ***p < 0.01, ***p < 0.001 compared with siNC group

As CIAPIN1 was overexpressed in lung cancer and might be a direct functional target of miR-195-5p in NSCLC cells, we next performed loss-of-function assays in A549 cells to further examine the biological function of CIAPIN1. Firstly, western blot analysis indicated that siCIAPIN1 transfection effectively down-regulated the protein expression of CIAPIN1 expression in A549 cells (Fig. 5a). Similarly, we found knockdown of CIAPIN1 significantly suppressed cell proliferation by MTT assay (Fig. 5b, *p* < 0.001). Furthermore, knockdown of CIAPIN1 could significantly arrested cell cycle at G0/G1 phase (Fig. 5c, *p* < 0.001) and promote cell early and late apoptosis (Fig. 5d, *p* < 0.01, *p* < 0.001).

## Discussion

The present study provided evidence that miR-195-5p is significantly downregulated in NSCLC tissues and cell lines and that miR-195-5p acts as tumor suppressor in NSCLC in vitro. Moreover, we found miR-195-5p has a tendency to associate with tumor size, TNM stage and lymph node metastasis. MiR-195-5p deficiency is an independent unfavorable prognostic factor for patients with NSCLC.

Indeed, it was reported that miR-195-5p was low-expressed in melanoma [27], oral squamous cell carcinoma [28], colon cancer [29], and hepatocellular carcinoma [30]. These studies revealed that miR-1301–3p plays a fundamental role in malignant suppression, and our results are consistent with these findings. NSCLC patients with tumors expressing low profiles of miR-195-5p exhibit poorer survival outcome. Thus, decreased miR-195-5p expression may present effective biomarker for prediction of a poor prognosis in NSCLC patients.

Further investigation demonstrated that restoration of miR-195-5p led to inhibition of NSCLC cells proliferation, and induction of G0/G1 cell arrest and apoptosis. We supposed that overexpression of miR-195-5p may attenuate cells viability though stalling the cells in G0/G1 phase and accelerating apoptosis. Importantly, several miRNAs have entered human clinical trials and miRNA-directed biological therapeutic agents is under way [31]. MiR-122 is associated with markedly clinical efficacy in phase I in hepatisis, has now reached phase II [32]. miR-34, known as anti-oncomiR, is being applied in phase I trials for defeating cancer [32]. In this study, the significant relationship between high miR-195-5p profiles with established less aggressive tumor biology indicating a potential therapeutic significance of miR-195-5p in NSCLC development and progression.

Furthermore, our studies revealed that the anti-apoptotic molecule CIAPIN1 is a direct target of miR-195-3p-mediated translational suppression in NSCLC cells. Oncomine microarray database showed that CIAPIN1 is expressed in high levels in lung adenocarcinoma tissues compared to normal lung tissues. A function for CIAPIN1 in NSCLC was further evaluated in vitro, confirming that depletion of CIAPIN1 phenocopied the effects of miR-195-5p on NSCLC cells behaviors. These studies suggest that down-regulation of miR-195-5p involved in the pathogenesis of NSCLC are partially alleviated by overexpression of CIAPIN1. Actually, the role of CIAP1N1 as an oncogene in tumorigenesis has recently described in various types of cancer, such as hepatocellular carcinoma [33], gastric cancer [34], and ovarian serous carcinoma [35]. Whereas, contrary to these reports, Chen et al. [36], Wang et al. [18] and Zheng et al. [37] have found that CIAPIN1 acts as a tumor suppressor in pancreatic cancer, clear cell renal cell carcinoma, and esophageal squamous cell carcinoma. Our results demonstrated a clear role of CIAPIN1 in the prevention of apoptosis and promotion of NSCLC cells growth. However, additional investigations to explore the molecular mechanisms of the aberrant expression of miR-195-5p in the metastasis and carcinogenesis of NSCLC are still needed.

In conclusion, this study principle finding is that low expression of miR-195-5p, a significantly dysregulated miRNAs in NSCLC, is associated with tumorigenesis and poor survival outcome in NSCLC patients, indicating that miR-195-5p may serve as a critical diagnostic and prognostic molecular marker. Moreover, restoration of miR-195-5p inhibited cell proliferation, and induced G0/G1 phase arrest and apoptosis via directly targeting CIAPIN1. This study greatly enriched our knowledge of the pathogenesis of NSCLC and may provide a novel therapeutic strategy for fighting against this disease.
